# Rethinking metabotropic glutamate receptor 5 pathological findings in psychiatric disorders: implications for the future of novel therapeutics

**DOI:** 10.1186/1471-244X-14-23

**Published:** 2014-01-28

**Authors:** Kelly A Newell, Natalie Matosin

**Affiliations:** 1 Centre for Translational Neuroscience, Faculty of Science, Medicine and Health and Illawarra Health and Medical Research Institute, University of Wollongong, Wollongong, NSW 2522, Australia; 2 Schizophrenia Research Institute, Darlinghurst, NSW 2010, Australia

**Keywords:** Metabotropic glutamate receptor, mGlu, mGluR5, GRM, Schizophrenia, Depression, Post-mortem human brain, Novel antipsychotic, Novel antidepressant, Glutamate

## Abstract

**Background:**

Pharmacological modulation of metabotropic glutamate receptor 5 (mGluR5) is of marked interest as a novel therapeutic mechanism to treat schizophrenia and major depression. However, the status of mGluR5 in the pathophysiology of these disorders remains unknown.

**Discussion:**

The majority of studies in the schizophrenia post-mortem brain indicate that total mGluR5 expression is unaltered. However, close examination of the literature suggests that these findings are superficial, and in actuality, a number of critical factors have not yet been considered; alterations may be highly dependent on brain region, neuronal population or molecular organisation in specific cellular compartments. A number of genetic knockout studies (mGluR5, Norbin, Homer1 etc.) continue to lend support to a role of mGluR5 in the pathology of schizophrenia, providing impetus to explore the regulation of mGluR5 beyond total mGluR5 protein and mRNA levels. With regards to major depression, preliminary evidence to date shows a reduction in total mGluR5 protein and mRNA levels; however, as in schizophrenia, there are no studies examining mGluR5 function or regulation in the pathological state. A comprehensive understanding of mGluR5 regulation in major depression, particularly in comparison to schizophrenia, is crucial as this has extensive implications for mGluR5 targeting novel therapeutics, especially considering that opposing modulation of mGluR5 is of therapeutic interest for these two disorders.

**Summary:**

Despite the complexities, examinations of post-mortem human brain provide valuable insights into the pathologies of these inherently human disorders. It is important, especially with regards to the identification of novel therapeutic drug targets, to have an in depth understanding of the pathophysiologies of these disorders. We posit that brain region- and cell type-specific alterations exist in mGluR5 in schizophrenia and depression, with evidence pointing towards altered regulation of this receptor in psychiatric pathology. We consider the implications of these alterations, as well as the distinction between schizophrenia and depression, in the context of novel mGluR5 based therapeutics.

## Background

Metabotropic glutamate receptor subtype 5 (mGluR5) is an exciting novel drug target for the treatment of psychiatric disorders including schizophrenia and major depression [1,2]. While the monoaminergic systems (e.g. dopamine, serotonin, norepinephrine) are the main therapeutic targets of current drugs used to treat schizophrenia and major depression, evidence suggests that the glutamatergic system and the N-methyl-D-aspartate receptor (NMDAR) in particular, play a key role in the underlying pathophysiology of these disorders, at least in a subset of patients [3-6]. Targeting the glutamatergic NMDAR is therefore considered a novel treatment strategy for these disorders. Accordingly, NMDAR agonists such as d-serine show therapeutic potential for the treatment of schizophrenia [7], whilst the NMDAR antagonist ketamine shows rapid antidepressant effects in patients with treatment-resistant depression [8,9]. In both cases however, NMDAR-targeting drugs are confounded, specifically by high dose requirements and the associated risk of excitotoxicity in the case of agonists [7], and psychotomimetic effects, addictive properties and cognitive decline in the case of antagonists [10-13]. As a result of these confounds, the metabotropic glutamate receptors have been identified as a possible avenue to regulate glutamatergic transmission, while minimising the unwanted effects of targeting the NMDAR directly [2,14]. mGluR5 is of particular interest as it shares a structural and functional link with the NMDAR, but unlike the NMDAR it is localised in more selective brain regions where it fine-tunes glutamatergic transmission [1].

mGluR5 positive allosteric modulators (PAMs) upregulate NMDAR function [15], whilst mGluR5 antagonists and negative allosteric modulators (NAMs) downregulate NMDAR activity [16]. These agents therefore have the potential to treat NMDAR hypofunction and hyperfunction states associated with schizophrenia and major depression respectively. In spite of this exciting possibility, the success of novel mGluR5-based therapeutics is dependent on the status of mGluR5 in these disorders. Conflicting data between human pathophysiological studies, genetic studies and animal studies make it unclear whether mGluR5 is indeed altered in these pathologies and what implications this has for novel mGluR5 based therapeutics.

## Discussion

### Neuropathological findings and considerations

#### Protein studies

Post-mortem analyses of human brain tissue from psychiatric patients provide valuable insight into the potential pathophysiology of these disorders. Often however, findings in post-mortem human brain are inconsistent with findings in more mechanistic studies such as animal- and/or cell- based studies, highlighting the complexities of these psychopathologies. We have recently reviewed the literature with regards to examination of mGluR5 in schizophrenia [1]. Three out of four studies examining mGluR5 protein in post-mortem brain samples from schizophrenia subjects (number of subjects ranging from 9 to 37) identified no change in mGluR5 protein (monomer) levels in the prefrontal cortex (PFC), specifically Brodmann’s areas (BA) 9, 10, 11, 32 and 46 as well as the caudate, putamen and nucleus accumbens [17-19]. In contrast to these findings, Fatemi and colleagues [20] recently reported a large reduction in mGluR5 protein (monomer) levels in the PFC (BA9) and lateral cerebellum of subjects with schizophrenia compared to controls (n = 15-20). Collectively, these findings raise the strong probability that alterations to mGluR5 in schizophrenia are brain region-specific and possibly cohort specific. This reinforces the importance of replication of findings in adjacent brain regions and independent cohorts and raises the notion that it is likely that mGluR5-based therapeutics will mediate region-specific effects as well as individual-specific effects depending on the underlying neuropathology.

In major depression, mGluR5 monomer protein levels have been reported to be reduced in the lateral cerebellum [20] and prefrontal cortex (BA10) [21] using two different cohorts (n = 15). While this might suggest less heterogeneity of changes in this disorder, the findings should be deemed as preliminary until additional brain regions are examined and findings further replicated in independent cohorts. An important way forward for both schizophrenia and major depression will be to identify these cohort specific changes in mGluR5, as well as other markers, in living patients. The detection of peripheral biomarkers, such as single-nucleotide polymorphisms, that have associations with symptoms and brain pathology (for example, see [5,22]), may pave the way for an individualised treatment approach.

A fundamental issue confounding the interpretation of these aforementioned immunoblot studies is that the majority have focused on mGluR5 in the monomer form. Mature functional mGluR5s exist in a disulphide-linked dimer complex [23,24] and can only be activated by agonists when in this dimer form [23]. Thus, alterations in monomers alone may not have functional significance. While one study reported no change in mGluR5 dimer levels in the PFC (BA9) and lateral cerebellum in schizophrenia and major depression [20], the use of reducing conditions (5% β-mercaptoethanol, which breaks disulphide links) would have interfered with the dimerized status of mGluR5. It is therefore unlikely that total dimers were indeed measured and rather these measures were an artefact of the experimental procedure, whereby the ratio of monomers and dimers is reflective of the amount of reducing agent used rather than a physiological indication. There are no reported investigations of mGluR5 dimer measures in non-reducing conditions in schizophrenia or depression, however altered dimerization of another mGluR, mGluR3, has been found in the PFC of schizophrenia subjects [25]. It is crucial to take these experimental variables into account for future mGluR5 immunoblot studies, and to determine whether dimer assembly is disrupted in these neuropathologies, as this would render mGluR5 present, but inactive, thereby having implications for the novel class of mGluR5-targeting therapeutics. While mGluR5 monomers are considered non-functional as they cannot be activated by agonists, a recent report suggests that mGluR5 monomers are able to couple to G-proteins upon activation with a PAM [23]. However more research is required to determine if mGluR5 dimers are altered in these psychiatric pathologies and what impact this has on the effectiveness of mGluR5 allosteric agents [26].

#### Receptor binding studies

Another valuable approach to understanding mGluR5 in psychiatric pathology and its potential as a therapeutic target is the use of radiolabeled drugs that target the therapeutic binding site of interest. We have recently reported no change in binding to the MPEP site of mGluR5 in the post-mortem dorsolateral PFC of schizophrenia patients [17]. We have also since found unaltered mGluR5 binding in the anterior cingulate cortex of schizophrenia, major depression and bipolar subjects in the Stanley Neuropathology Consortium (unpublished observations). Considering many novel mGluR5 drugs target the MPEP site, our findings suggest that this binding site is accessible in these disorders, at least in the dorsolateral PFC and anterior cingulate cortex. While these are the only studies to have examined mGluR5 binding in schizophrenia, Deschwanden et al. [21] reported region-specific reductions in [^11^C]ABP688 in-vivo binding to the MPEP site in the PFC, insula, and parts of the parietal and temporal cortices in living patients with depression. These alterations were associated with depressive symptom severity. Considering Deschwanden and colleagues also reported reductions in mGluR5 protein in the PFC in major depression (in a separate post-mortem cohort) [21], it is possible that these reductions in binding and protein represent a reduction in overall mGluR5 numbers in the PFC in depression. Notably, in line with our observations, no significant change in binding was reported in the anterior cingulate cortex [21]. The assessment of binding specifically to membrane bound mGluR5 and the affinity of novel mGluR5 targeting drugs to binding sites in the brains of patients with schizophrenia and depression will aid in understanding the potential of these drugs for the treatment of psychiatric disorders. While assessment of the MPEP binding site offers insight into the binding kinetics of mGluR5 drugs that target this site, it must be noted that there is a family of mGluR5 allosteric modulators that do not bind to this site [27-29], and currently there are no radioligands available to directly assess the density and affinity of these non-MPEP sites.

#### mRNA studies

Although mRNA analyses do not directly equate to functional protein expression [30], examination of mRNA offers several advantages. Firstly, it allows for analyses of amino acid sequences that are highly specific to the molecule or isoform of interest. Secondly, to gain an understanding of underlying mechanisms of any changes in protein expression (for example whether they may be the result of changes in rate of synthesis), examination of mRNA expression is essential. Most recently, it was reported that there was no change in mGluR5(pan) mRNA in the PFC(BA9), together with a reduction in mGluR5(pan) mRNA in the lateral cerebellum in schizophrenia [20]. This same study found reduced mGluR5 protein in both the cerebellum and PFC, indicating possible deficits in mGluR5 synthesis in the cerebellum, but post-transcriptional modifications in the PFC. Consistent with this, four previous studies found no change in mGluR5 mRNA in the PFC (BA9/10), as well as the thalamus or hippocampus in schizophrenia [31-34]. One study did however report an increase in mGluR5 mRNA expression in the PFC (BA11; n = 7-10), which was specific to cortical layer III [34], a layer concerned with cortico-cortical projections and that is particularly implicated in schizophrenia pathophysiology [35]. It should therefore be considered that alterations in mGluR5 mRNA are not only brain region-specific, but may also exist specifically in selective neuronal populations in the diseased brain, and analyses of total neuron populations may therefore conceal pathological alterations. With regards to major depression, preliminary studies suggest concordant reductions in mRNA and protein in post-mortem brain samples. Fatemi et al. [20] recently reported a reduction in mGluR5(pan) mRNA in the lateral cerebellum in major depression, consistent with their finding of reduced mGluR5 protein in this same region. This suggests that a reduction in mGluR5 production may occur, at least in this brain region, in major depression. To our knowledge, there are no other studies examining mGluR5 mRNA in major depression to shed light on the mechanism and extent of mGluR5 alterations in this disorder.

#### Further considerations

When approaching mGluR5 post-mortem findings in the context of future therapies, there has been minimal consideration of the structural complexity of this receptor. To date, mGluR5a, b, and d isoforms have been identified. These isoforms differ in their developmental profile, brain region localisation, and length of their c-terminus [1,36,37]. While mGluR5a and mGluR5b share similar pharmacological profiles they have opposing functions, at least in development, where mGluR5a inhibits and mGluR5b promotes neurite outgrowth [37]. Compared to mGluR5a and b, the mGluR5d isoform has increased sensitivity to desensitisation and has a shorter intracellular c-terminus by a significant 267 amino acids [36]; consequently mGluR5d has less interaction with scaffolding molecules that modulate the mGluR5/NMDAR complex (e.g. Homer), and other endogenous molecules that ensure its trafficking and cell surface localisation. This is important to consider, especially in the context of schizophrenia where the majority of studies indicate no change in overall mGluR5 levels; there may in reality be altered regulation, trafficking or recycling of mGluR5 or altered receptor-receptor interactions. For example, Homer, Norbin (neurochondrin) and Tamalin (GRASP1) act as chaperones to increase cell surface localisation of mGluR5. Homer and Norbin knockout mice show a schizophrenia-like behavioural phenotype [38,39], suggesting there may be deficits in membrane-bound localisation of mGluR5 in schizophrenia. Supporting this hypothesis, the mGluR5 *GRM5* gene has recently been suggested as one of the top candidate genes for schizophrenia vulnerability [40,41], with exome sequencing of multiplex pedigrees reporting disruption to the mGluR5/Tamalin association [40]. Consistent with this notion, mGluR5 knockout mice and mice treated with mGluR5 antagonists also demonstrate schizophrenia-like behaviours (and interestingly antidepressant behaviours, as discussed below) [1,42]. While these mGluR5 regulatory molecules have not been considered in the context of depression, *Homer1* has been implicated in the aetiology of major depression through a genome wide association study [43], suggesting possible disruptions to mGluR5 trafficking and/or mGluR5/NMDAR interactions in this disorder also.

### Opposing glutamatergic dysregulation in schizophrenia and depression: Implications for novel mGluR5 therapeutics

mGluR5 PAMs have shown promise in preclinical rodent models for the treatment of schizophrenia [1]. mGluR5 PAMs, such as CDPPB and more recently VU0364289, demonstrated the ability to attenuate phencyclidine and amphetamine-induced hyperlocomotion, social interaction deficits, prepulse inhibition deficits and importantly, cognitive deficits (which are largely untreated by current antipsychotics) [1,44]. While the development of mGluR5 PAMs have faced issues with regards to solubility and oral bioavailability, a new generation of PAMs appear to have overcome these issues [45-47]. In contrast, mGluR5 NAMs, including MPEP and MTEP, have shown antidepressant properties in rodent models, including reduced immobility time in forced swim and tail suspension tests, key tests of antidepressant efficacy in rodent models [48-50]. However, some mGluR5 NAMs have also shown the ability to produce psychotomimetic side effects in rodent models, similar to the NMDAR antagonist ketamine [51]. More recent drug design has resulted in the development of mGluR5 NAMs that appear to lack this psychotomimetic property [47].

While the glutamatergic system is implicated in the pathophysiologies of both schizophrenia and major depression, the use of mGluR5 PAMs for the treatment of schizophrenia and NAMs for the treatment of depression, suggests opposing disturbances of the glutamatergic system in these disorders. Although the data for mGluR5 in the schizophrenia brain is conflicting across studies [1,20] (possibly as a result of region-specific pathology), a decline in mGluR5 in depression has now been replicated in three cohorts [20,21]. However, negative modulation of mGluR5 has been reported to have therapeutic efficacy in preclinical (rodent) models of depression [9,52]. In addition, NMDAR antagonists, such as ketamine, demonstrate antidepressant properties in treatment resistant patients [10]. From the outset, the use of these therapies might indicate a hyperglutamatergic state or specific NMDAR hyperfunction in depression, suggesting that the mGluR5 reduction seen in patients with depression represents an endogenous compensatory response, and that the use of mGluR5 NAMs may act to further assist this mGluR5 downregulation. This is supported by findings of increased glutamate levels in the brains of some patients with depression [4,53-55]. As the results of clinical trials for mGluR5 NAMs in depression are not yet available, the therapeutic effects remain to be seen. It is likely that the incorporation of mGluR5-based therapeutics will be individualized depending on symptom profile and individual pathology.

The issues raised here are not limited to schizophrenia and major depression. Glutamatergic dysfunction is also implicated in other neuropsychiatric disorders including anxiety and Fragile X Syndrome. mGluR5 NAMs prevent behavioural phenotypes in animal models of these disorders [56-58]. mGluR5 NAMs including Fenobam and AFQ056 have progressed to clinical trials where they showed therapeutic effects for the treatment of anxiety and Fragile X Syndrome [59-61]. However there were reports of psychotomimetic effects following chronic treatment in some patients [59]. In addition, a recent study provides evidence that patient response to these mGluR5 NAMs may depend on individual pathophysiology [61]. This demonstrates, despite some limitations which are being considered in current drug design [62], that this class of drugs has the potential to progress into clinical therapeutics. mGluR5 PAMs on the other hand are yet to reach clinical trials.

## Summary

We suggest that, despite post-mortem studies largely indicating no involvement of mGluR5 in the pathology of schizophrenia, and preliminary evidence suggesting it is down-regulated in major depression, that there is evidence in both pathologies of altered regulation of this receptor, possibly in a brain region- and cell type-specific manner. In spite of the rapid movement to develop mGluR5 pharmaceutical modulators, there has been very limited investigation into the function and regulation of mGluR5 at the genetic, mRNA and protein levels in the pathological post-mortem human and animal paradigms [63]. Only with this knowledge will we be able to tailor novel therapeutics to treat these diseases. It is likely that mGluR5-based therapeutics will be much individualised depending on symptom profile. Pharmacogenomics or peripheral biomarkers may help to identify the patients that would suit mGluR5-modulation as a therapy option.

## Abbreviations

BA: Brodmann’s area; mGluR5: Metabotropic glutamate receptor subtype 5; NAM: Negative allosteric modulator; NMDAR: N-methyl-D-aspartate receptor; PAM: Positive allosteric modulator; PFC: Prefrontal cortex.

## Competing interests

The authors declare that they have no competing interests.

## Authors’ contributions

KAN and NM drafted the manuscript. Both authors read and approved the final manuscript.

## Authors’ information

KAN is a Senior Lecturer in the Faculty of Science, Medicine and Health, at the University of Wollongong, Australia. NM is a doctoral candidate in the Faculty of Science, Medicine and Health, at the University of Wollongong, Australia. KN and NM are both affiliated scientists of the Schizophrenia Research Institute, Australia. NM is supported by an Australian Rotary Health Ian Scott Scholarship.

## Pre-publication history

The pre-publication history for this paper can be accessed here:

http://www.biomedcentral.com/1471-244X/14/23/prepub
